# Exploring the Molecular Modalities in the Pathogenesis of Diabetic Kidney Disease with a Focus on the Potential Therapeutic Implications

**DOI:** 10.3390/biomedicines13010050

**Published:** 2024-12-28

**Authors:** Lyubomir Gaydarski, Kristina Petrova, Ivan Angushev, Stancho Stanchev, Alexandar Iliev, Nikola Stamenov, Vidin Kirkov, Boycho Landzhov

**Affiliations:** 1 Department of Anatomy, Histology and Embryology, Medical University of Sofia, 1431 Sofia, Bulgaria; kristinapetrova270@gmail.com (K.P.); iangushev@gmail.com (I.A.); stanchev_1989@abv.bg (S.S.); dralexiliev@abv.bg (A.I.); nikola.stamenov1@gmail.com (N.S.); landzhov_medac@abv.bg (B.L.); 2 Department of Health Policy and Management, Faculty of Public Health ‘Prof. Dr. Tzekomir Vodenicharov’, Medical University of Sofia, 1527 Sofia, Bulgaria; vidinkk@abv.bg

**Keywords:** diabetic kidney disease, apelin, apelin receptor, vascular endothelial growth factor, vascular endothelial growth factor receptors, nitric oxide, nitric oxide synthase

## Abstract

Diabetic kidney disease (DKD) is a leading cause of chronic kidney disease (CKD) and end-stage renal disease worldwide, affecting approximately 40% of individuals with type 2 diabetes (T2DM) and 30% of those with type 1 diabetes (T1DM). As the prevalence of diabetes continues to rise, the burden of DKD is expected to grow correspondingly. This review explores the roles of key molecular pathways, including the apelinergic system, vascular endothelial growth factor (VEGF)/VEGF receptor (VEGFR) axis, and nitric oxide (NO)/nitric oxide synthase (NOS) signaling, in DKD pathogenesis and potential therapeutic applications. The apelinergic system, involving apelin and its receptor (APLNR), influences endothelial function, glucose metabolism, and renal health. Preclinical studies highlight its dual role in renal protection and injury through anti-inflammatory and antioxidant pathways, while other evidence suggests that it may exacerbate DKD through podocyte damage and angiogenesis. Similarly, the VEGF/VEGFR axis demonstrates a complex contribution to DKD, where VEGF-A promotes pathological angiogenesis and glomerular damage, but its inhibition requires careful modulation to prevent adverse effects. The NO/NOS system, integral to vascular and renal homeostasis, also exhibits altered activity in DKD, with reduced bioavailability linked to oxidative stress and inflammation. This review underscores the intricate interplay between these pathways in DKD, revealing both challenges and opportunities in their therapeutic targeting. Further research is essential to refine strategies and develop effective interventions for DKD management.

## 1. Introduction

Chronic kidney disease (CKD) is characterized by the presence of albuminuria and a reduced estimated glomerular filtration rate (eGFR) [1]. CKD is becoming increasingly prevalent, affecting an estimated 840 million people worldwide, or approximately 10% of the global population [2,3]. Diabetic kidney disease (DKD), which results from diabetes-induced kidney damage, is the leading cause of CKD, impacting nearly 40% of individuals with type 2 diabetes mellitus (T2DM) and approximately 30% of those with type 1 diabetes mellitus (T1DM) [1,3]. Given the rising global prevalence of diabetes, the number of people living with DKD is expected to increase significantly [3].

This paper provides a narrative review that does not involve a systematic search of the included references. It focuses on the roles of three molecular systems—the apelinergic system, the vascular endothelial growth factor (VEGF)/VEGF receptor (VEGFR) axis, and the nitric oxide (NO)/nitric oxide synthase (NOS) signaling pathways—in the pathogenesis of DKD. By examining these systems, the review aims to elucidate their complex involvement in the intricate adaptive mechanisms underlying DKD progression and to explore their potential as targets for future therapeutic interventions.

## 2. Role of the Apelinergic System in DKD

### 2.1. Overview

The apelinergic system consists of a G-protein-coupled receptor (APLNR) and two endogenous peptide ligands, apelin and elabela [4]. This system is pivotal in maintaining energy homeostasis, brain function and involves various physiological processes, including cardiovascular regulation and renovascular homeostasis [5,6,7,8]. In recent years, apelin signaling through the APLNR receptor in endothelial cells has garnered attention as a potential therapeutic target for DKD and other metabolic disorders [9].

Apelin is particularly important for endothelial function, with significant roles in regulating glucose metabolism and lipid transport. Research indicates that apelin signaling influences endothelial glucose uptake, enhances insulin sensitivity, and modulates fat accumulation by regulating the FOXO1 transcription factor [9]. These findings underscore the therapeutic potential of the apelinergic system in addressing the metabolic dysregulation underlying DKD and related conditions.

### 2.2. Animal Studies

Apelin is expressed in the pancreatic islets of various species, including humans, mice, rats, pigs, and cats, with its expression regulated by glucocorticoids rather than glucose [10]. Hyperglycemia, a hallmark of diabetes, disrupts glomerular hemodynamics, leading to glomerular hypertrophy, damage to the filtration barrier, and albuminuria. Evidence suggests that the apelinergic system is dysregulated in DKD [10,11]. For example, in cultured mouse podocytes, high glucose levels downregulate APLNR mRNA expression [11] but simultaneously upregulate APLNR protein expression [12]. This dysregulation is further exacerbated by angiotensin II (Ang II), which amplifies the downregulation of APLNR mRNA [11]. In some T1DM and T2DM models, the reduced expression of APLNR has been observed, while others report increased expression, reflecting variability in the apelinergic system’s role in DKD pathogenesis [11,13]. Functionally, apelin-13 exhibits protective effects in specific contexts, such as cultured mouse podocytes, demonstrating anti-apoptotic properties [11]. Similarly, in T1DM mouse models, apelin-13 preserves glomerular structure, reduces proteinuria, and suppresses renal inflammation [14]. Elevated apelin levels in rats indicate a significant anti-T2DM effect, highlighting its role as an insulin-sensitizing agent [15]. In mice, the administration of apelin-13 has been shown to lower blood glucose levels via an eNOS-dependent mechanism, enhancing glucose turnover [15]. In diabetes-related complications such as diabetic retinopathy and nephropathy, apelin-13 levels are significantly higher in diabetic tissues compared to non-diabetic ones [16]. Studies on diabetic rat kidneys have demonstrated that apelin-13 administration can restore the expression of catalase, an antioxidant enzyme typically downregulated in diabetic conditions, suggesting a potential renoprotective effect through its antioxidant properties [17]. Gao et al. examined the role of apelin-13 in DKD using rat models, finding promising results [18]. The administration of apelin-13 over 8 weeks resulted in reduced weight and blood glucose levels while increasing insulin levels, indicating improved metabolic regulation. Kidney function markers showed improvement, reflecting reduced renal injury. The study highlighted apelin-13’s role in vascular regulation by the modulation of NO, Ang II, and endothelin-1 production, as assessed via Western blotting. Moreover, apelin-13 positively influenced the expression of fibrosis-related proteins such as E-cadherin and α-SMA, suggesting a role in mitigating renal fibrosis. These findings underscore apelin-13’s potential protective effects in DKD through its involvement in the apelinergic system and associated signaling pathways [18].

However, apelin’s effects are not uniformly beneficial. In diabetic mice with nephropathy, apelin promotes podocyte apoptosis, which appears to be inversely related to podocyte autophagy [16]. This effect is likely mediated through the mTOR pathway, which has been identified as a key mechanism by which apelin inhibits podocyte autophagy [16]. Furthermore, in diabetic retinopathy, apelin-13 has been implicated in promoting retinal neovascularization, further illustrating its dual role in diabetes-related complications [17]. In the KK-Ay mouse model of T2DM, apelin appears to exacerbate disease progression, worsening albuminuria and creatinine clearance. These adverse effects are attributed to disruptions in renal blood flow, increased podocyte apoptosis, and impaired autophagy [12,13]. Liu et al. investigated the role of apelin in DKD, with a specific focus on its effects on podocyte function and autophagy [19]. Their study revealed that apelin treatment in diabetic mice exacerbated renal dysfunction, evidenced by an increased urinary albumin-creatinine ratio and reduced creatinine clearance. Apelin aggravated podocyte injury, leading to foot process effacement and apoptosis, while the apelin antagonist F13A mitigated these harmful effects. Mechanistically, apelin’s detrimental impact was attributed to suppressed autophagy in podocytes, as reflected by the decreased expression of autophagy-related proteins, including LC3, Beclin-1, and Atg5. This suppression was mediated via the activation of the mTOR pathway, which was further stimulated by apelin but counteracted by F13A. Complementary in vitro studies confirmed that apelin inhibits podocyte autophagy and promotes apoptosis through the mTOR signaling pathway, ultimately contributing to the progression of DKD [19]. This dualistic role of apelin highlights the complexity of its involvement in DKD, emphasizing the need for further investigation into its mechanisms and therapeutic potential.

Knockout models of APLNR in endothelial cells exhibited impaired glucose utilization, elevated blood glucose levels, and excessive fatty acid (FA) accumulation in essential metabolic tissues, highlighting the critical role of endothelial apelin/APLNR signaling in maintaining glucose and lipid homeostasis [9]. Furthermore, inhibiting FABP4, a fatty acid-binding protein, reversed these metabolic abnormalities, identifying it as a potential downstream mediator of apelin’s effects. These findings underscore the therapeutic potential of targeting apelin signaling to enhance vascular and metabolic health in DKD [9]. When APLNR expression was selectively knocked out in endothelial cells, apelin treatment failed to activate key downstream metabolic signaling pathways, including AKT and AMPK phosphorylation, which are typically active in these tissues [9]. In vivo studies revealed that mice lacking endothelial APLNR displayed impaired glucose tolerance, reduced insulin sensitivity, and increased FA accumulation in metabolically active tissues [9]. Interestingly, apelin regulates metabolic processes through the transcription factor FOXO1 and downstream targets such as FABP4, which mediate FA uptake and transport across endothelial cells [9]. The inhibition of FABP4 in APLNR-deficient mice restored glucose utilization and normalized FA uptake, emphasizing the potential of targeting the endothelial apelin/FOXO1/FABP4 axis to improve metabolic health in DKD. These insights provide a robust foundation for developing apelin-based therapies to manage DKD-associated metabolic dysfunction [9].

### 2.3. Human Studies 

In a comprehensive study, Nyimanu et al. utilized autoradiography, immunohistochemistry, and ELISA to analyze the expression of apelin, ELA, and APLNR in the normal human kidney [20]. Immunofluorescence demonstrated that apelin, ELA, and APLNR colocalized with von Willebrand factor in intrarenal arteries and arterioles, while their expression was also observed in vascular smooth muscle cells [20]. Interestingly, these proteins were localized to epithelial cells within the nephron, particularly in the glomerulus, with limited expression in the endothelium. This suggests potential expression by other cell types, such as podocytes. Apelin and ELA were detected on the apical and basolateral membranes of epithelial cells in the proximal convoluted tubule (PCT), with apelin localized explicitly at the apical membrane [20]. The apelinergic system also colocalized with SGLT2, indicating a potential role in glucose handling within the kidney. Apelin, ELA, and APLNR were expressed throughout the loop of Henlé. In the distal convoluted tubule, the apelin receptor colocalized with TRPM6, a magnesium channel [20]. Notably, the presence of APLNR in the juxtaglomerular apparatus was revealed by its co-localization with renin. Additionally, all three components—apelin, ELA, and APLNR—were identified in the epithelial cells of the collecting duct, where they colocalized with aquaporin 4 in principal cells. The co-localization of APLNR and SGLT2 in the PCT is particularly intriguing [20]. Since apelin is known to regulate glucose homeostasis and enhance insulin sensitivity, Nyimanu et al. propose that apelin may influence the SGLT2 pathway, promoting glucose excretion by the kidney. This mechanism could offer therapeutic benefits for patients with DKD [20]. 

Habchi et al. evaluated serum apelin levels across a cohort comprising patients with T1DM and T2DM and non-diabetic controls, examining their relationship with glycemic control [21]. The study included 130 T1DM patients, 98 T2DM patients, and 162 healthy controls, with participants carefully screened to exclude acute or chronic illnesses and specific treatments [21]. Metabolic and lipid profiles and serum apelin levels were assessed using standardized laboratory methods [21]. The results revealed distinct differences in serum apelin levels among the groups [21]. Diabetic patients exhibited higher apelin levels than controls, with T1DM patients showing the highest concentrations [21]. In the control group, apelin levels positively correlated with BMI, age, and LDL cholesterol but showed no significant associations with glucose or triglycerides [21]. In T2DM, apelin correlated negatively with BMI and HbA1c, suggesting a role in glycemic regulation. However, no such associations were observed in T1DM [21]. Notably, poorer glycemic control (HbA1c ≥ 7.5%) was linked to lower apelin levels in T1DM, although this correlation was weaker than in T2DM [21]. Overall, this study highlights a differential role of apelin in diabetes, particularly in its potential involvement in metabolic pathways and glycemic control, with a more pronounced association observed in T2DM [21].

A population-based study in China investigated associations between apelin gene single nucleotide polymorphisms (SNPs)—rs2281068, rs3115757, rs2235309, and rs2235310—and T2DM in a cohort of 498 patients and 1,468 controls. Initial analyses found no direct correlation between these SNPs and T2DM, although a strong linkage disequilibrium was observed between rs3115757 and rs2281068 [22]. Further analysis identified a significant association between rs2281068 and T2DM, with rs3115757 excluded due to a violation of Hardy–Weinberg equilibrium [22]. Comparisons based on gender and genotype-clinical parameters revealed no significant findings. These results suggest that rs2281068 is a key genetic factor in T2DM, providing new insights into the apelinergic system’s potential involvement in DKD pathogenesis [22].

Kotanidou et al. investigated serum apelin levels in obese children and adolescents, discovering significantly lower levels in the obese group than healthy controls [23]. Apelin levels were inversely correlated with BMI and markers of insulin resistance, such as HOMA-IR. Among the obese participants, 71.1% were diagnosed with prediabetes, and those with prediabetes had markedly lower apelin levels than healthy controls. The analysis suggested that apelin levels might influence the likelihood of prediabetes; however, this association lost significance after adjusting for BMI and HOMA-IR. Additionally, the study examined the prevalence of the G212A polymorphism in the APLNR gene, finding that participants with the AA genotype had higher apelin levels than those with GG and GA genotypes. Overall, these findings underscore a potential role for the apelinergic system in metabolic dysregulation among obese youths, particularly in impaired glucose metabolism [23]. The findings from the discussed human studies are comprehensively summarized in Table 1.

As shown in this review, many fundamental animal studies have been conducted on the role of the apelinergetic system in the pathogenesis of DKD, yet few human studies have been conducted. Further research on the potential diagnostic and therapeutic properties of the apelinergetic system in humans with DKD is required.

### 2.4. Potential Treatment Implications

DKD is closely linked to metabolic disturbances and oxidative stress resulting from hyperglycemia, with chronic inflammation playing a pivotal role in its progression [17,24]. The role of the apelinergic system in the pathogenesis and progression of DKD remains controversial and not yet fully understood. Preclinical studies suggest that the apelinergic system has renoprotective potential by mitigating diabetes-induced renal damage, suppressing inflammation, and enhancing antioxidant pathways [17,24]. This system presents promising therapeutic opportunities for managing DKD through multiple mechanisms, including synergistic effects with RAAS inhibitors, direct renoprotective actions, and cardiovascular protection [25]. Combining apelin receptor agonists with RAAS inhibitors may further enhance renal protection and improve cardiovascular outcomes [25]. Additionally, apelin supports kidney function by promoting aquaresis, modulating glomerular pressure, and improving renal hemodynamics [25]. Given the frequent coexistence of DKD with cardiovascular complications, the cardioprotective properties of apelin offer a dual therapeutic benefit [25].

However, apelin demonstrates a dual role in DKD, exhibiting protective and potentially harmful effects depending on the context and underlying mechanisms. Protective effects, particularly of apelin-13, include reductions in kidney damage, inflammation, hypertrophy, and proteinuria and improvements in renal function in diabetic mice [17,25,26]. On a molecular level, apelin normalizes oxidative stress by upregulating catalase expression, reduces inflammation through decreased MCP-1 and NF-κB activation, and inhibits histone hyperacetylation by increasing HDAC1 expression [14,17]. Apelin signaling also addresses gaps left by traditional antidiabetic therapies by enhancing endothelial function and glycemic control [9]. Furthermore, apelin promotes renal hemodynamics by increasing blood flow and diuresis and upregulating antioxidant enzymes, countering the overactive RAAS commonly seen in CKD and DKD [25,27].

The co-localization of apelin with the SGLT2 receptor [20] suggests a potential interaction with SGLT2 inhibitors (SGLT2i). A systematic review by Lardaro et al. highlighted the anti-inflammatory properties of SGLT2i and their potential cognitive benefits in reducing dementia and cognitive decline [28]. While current studies on the cognitive effects of SGLT2i are limited and yield conflicting results, Lardaro et al. emphasized the need for further trials to evaluate their efficacy in treating Alzheimer’s disease and other cognitive disorders, intending to improve quality of life and slow disease progression [28]. Investigating the relationship between apelin and SGLT2i could advance treatment options for DKD and potentially extend therapeutic benefits to neurocognitive conditions.

Conversely, elevated apelin levels have been associated with adverse outcomes, including increased markers of renal fibrosis and dysfunction, such as TGF-β1 and urinary protein levels, indicating that high apelin levels may signal worsening kidney function or serve as biomarkers for advanced DKD [29]. Elevated apelin has been implicated in impairing podocyte function, increasing glomerular endothelial permeability, and promoting abnormal angiogenesis, which may exacerbate kidney damage [13]. Moreover, apelin’s involvement in promoting diabetic retinopathy underscores the need for caution to prevent complications in other organs [17,19]. These contradictory findings highlight the importance of further investigation to clarify the apelinergic system’s dual roles and therapeutic potential in DKD.

In conclusion, while the apelinergic system holds considerable promise as a therapeutic target for DKD, additional research is essential to elucidate its mechanisms of action and clinical potential fully. Future research should include clinical trials to evaluate the safety and efficacy of apelin receptor agonists in DKD patients and detailed mechanistic studies to understand better apelin’s influence on cellular pathways in diabetic nephropathy [25]. Long-term investigations are also necessary to determine whether targeting the apelinergic system improves kidney function and overall health outcomes [25].

## 3. Role of VEGF/VEGFR System in DKD

### 3.1. Overview

VEGF is a naturally occurring peptide critical in developing new blood vessels. In humans, five VEGF isoforms have been identified. VEGF-A, VEGF-B, and placental growth factor (PGF) are primarily involved in promoting the formation of new blood vessels, while VEGF-C and VEGF-D are more closely associated with lymphatic vessel development [30]. The VEGF family functions by binding to specific VEGF receptors (VEGFR), part of the tyrosine kinase receptor superfamily. There are three VEGFR subtypes, VEGFR1 and VEGFR2, predominantly expressed in endothelial cells, macrophages, monocytes, and hematopoietic cells, interacting with VEGF-A, VEGF-B, and PGF with high affinity to regulate angiogenesis [31,32]. In contrast, VEGFR3 is primarily expressed in the endothelial cells of lymphatic vessels and binds to VEGF-C and VEGF-D, playing a pivotal role in lymphangiogenesis [33].

### 3.2. Animal Models 

Angiogenesis involves complex interactions between angiogenic growth factors, endothelial cells (EC), and the extracellular matrix [34]. This process includes EC proliferation, migration, morphological changes, and matrix remodeling, orchestrated by a delicate balance between stimulatory and inhibitory factors [35]. The disruption of this balance can result in pathological angiogenesis [35]. Abnormal angiogenesis, such as the formation of atypical blood vessels around glomeruli in diabetic mouse models, has been implicated in the progression of diabetic kidney disease (DKD) [36]. Excessive and abnormal angiogenesis in the early stages of DKD leads to structurally immature and highly permeable capillaries [37]. These immature vessels contribute to plasma protein extravasation and an enlarged glomerular filtration surface, which result in hyperfiltration, a rapid decline in the glomerular filtration rate, and increased albuminuria [38]. Conversely, in advanced DKD, capillary rarefaction reduces oxygen and blood supply, exacerbating tubular cell loss and fibrosis [39]. Experimental models have demonstrated that VEGF-A overexpression induces glomerular hypertrophy, basement membrane thickening, and mesangial expansion, which are hallmark features of DKD [40,41]. Mesangial cells contribute to these processes, promoting angiogenesis through VEGF-A upregulation [42]. VEGF-A and VEGFR2 are critical mediators of angiogenesis and play significant roles in the pathophysiology of DKD [43]. VEGF-A is predominantly expressed in glomerular podocytes and tubular cells, while VEGFR2 is primarily localized in glomerular endothelial cells [44]. In early-stage DKD, VEGF-A expression is upregulated, activating a paracrine signaling pathway between podocytes and endothelial cells. This interaction promotes angiogenesis through the PI3K/Akt and ERK pathways [40].

Animal models of T1DM and T2DM consistently show the upregulation of VEGF-A and its receptors in the glomeruli [45,46,47]. In vitro studies demonstrate that high glucose levels directly enhance VEGF-A expression in podocytes and mesangial cells [48]. The podocyte-specific overexpression of VEGF-A in diabetic rats accelerates diabetic glomerulopathy, characterized by nodular glomerulosclerosis, microaneurysms, glomerular basement membrane (GBM) thickening, podocyte effacement, and significant proteinuria. These findings underscore the pathogenic role of local VEGF-A overexpression in DKD [40]. Conversely, inhibiting the VEGF/VEGFR pathway with anti-VEGF monoclonal antibodies reduces albuminuria, glomerular hypertrophy, and structural damage in diabetic animal models [49]. Similarly, VEGFR inhibitors have demonstrated renoprotective effects, mitigating structural and functional renal alterations [49].

Chyla-Danil investigated the effects of suramin on the VEGF-A/VEGFR axis and endothelial function in DKD using a short-term streptozotocin-induced diabetes rat model [50]. Diabetic rats exhibited reduced VEGF-A protein levels and altered endothelial responses. Suramin treatment did not significantly affect VEGF-A protein levels in isolated glomeruli but reduced VEGF-A expression in kidney tissue and notably altered its localization [50]. 

Cooper et al. (1999) explored the role of VEGF and VEGFR-2 in the kidneys of diabetic rats. The findings indicate that short-term diabetes led to a threefold increase in VEGF mRNA and a twofold increase in VEGFR-2 gene expression, while long-term diabetes resulted in a sustained twofold increase in VEGF but no significant change in VEGFR-2 expression. VEGF mRNA and protein were primarily localized in glomerular epithelial cells, distal tubules, and collecting ducts, with VEGFR-2 found in glomerular endothelial cells and cortical and renomedullary interstitial cells. Increased VEGF and VEGFR-2 expression in early diabetes correlated with heightened VEGF binding in glomeruli and renal medulla, suggesting an activation of the VEGF pathway. These results support VEGF’s involvement in diabetic kidney changes, although the long-term significance and effects of VEGFR-2 downregulation remain unclear [46]. Lavoz et al. explored the role of VEGFR2 signaling in DKD using the BTBR ob/ob mouse model, examining the effects of canonical ligands like VEGF and non-canonical ligands such as Gremlin [51]. The therapeutic inhibition of VEGFR2 activation was achieved with the kinase inhibitor SU5416, administered after the onset of DKD [51]. This treatment led to significant improvements, including reduced albuminuria, decreased renal structural damage, the attenuation of podocyte loss, and lower levels of inflammatory markers, indicating protection against DKD progression [51]. Histological and molecular analyses revealed less mesangial expansion, glomerular basement membrane thickening, and podocyte injury [51]. Inflammation was also mitigated, as evidenced by reduced immune cell infiltration and diminished expression of proinflammatory cytokines [51]. Interestingly, Gremlin expression was upregulated in diabetic kidneys at later stages of the disease, whereas VEGF-A expression peaked in the early stages before declining [51]. RNA sequencing further highlighted Gremlin’s role in regulating extracellular matrix-related genes and pathways central to DKD pathophysiology, providing valuable insights into VEGFR2-mediated mechanisms [51].

### 3.3. Human Studies 

In humans, VEGF-A levels are associated with the severity of albuminuria in DKD. However, the downregulation of VEGF-A has been observed in long-standing diabetes, suggesting its regulation varies across different stages of the disease [52]. This underscores the importance of refining VEGF-A modulation strategies to effectively target specific disease phases and identify patients most likely to benefit from VEGF-A inhibition therapies [52]. 

The overexpression of VEGF-A in diabetic patients is strongly linked to increased vascular permeability and the progression of nephropathy [53,54]. VEGFR1 has been implicated in regulating endothelial cell migration and proliferation, though its role in DKD is less clearly defined than VEGFR2 [55]. Studies suggest that while VEGFR1 and VEGFR2 are expressed in diabetic conditions, VEGFR2 levels show a stronger correlation with the severity of renal injury and vascular complications, highlighting its critical role in DKD pathophysiology [53]. The dysregulation of VEGF and its receptors in diabetes is closely associated with renal pathology and fibrosis, making them promising targets for therapeutic intervention. Evidence indicates that managing hyperglycemia and dyslipidemia can help mitigate the vascular complications of diabetes, underscoring the importance of maintaining a balance in angiogenic factors to prevent or delay the progression of DKD [53,56]. Renal biopsy studies reveal characteristic pathological changes, including mesangial expansion, abnormal capillary proliferation with thickened basal membranes, and smooth muscle layers, often resulting in plasma leakage [57]. While the exact role of angiogenesis in glomerular alterations remains unclear, studies suggest that inhibiting angiogenesis could slow DKD progression [42]. These findings highlight the significant contribution of abnormal angiogenesis to glomerular hypertrophy, basement membrane thickening, and mesangial expansion, supporting the further exploration of angiogenic pathways as potential therapeutic targets in DKD. Ruszkowska-Ciastek et al. investigated the concentrations of VEGF-A and its receptors, VEGFR1 and VEGFR2, in patients with well-controlled type 2 diabetes mellitus (T2DM) who exhibited no vascular complications [56]. The study included 31 diabetic patients aged 46–79, with HbA1c levels ≤6.5%, alongside a control group of 30 healthy individuals [56]. Vascular complications were ruled out through urinary albumin testing, retinal examination, and ankle–brachial index measurements. The diabetic participants received antihyperglycemic treatments (e.g., insulin, metformin, sulfonylureas), statins, ACE inhibitors, and occasionally antiplatelet or anticoagulant medications, excluding fibrinolytic agents [56]. Fasting venous blood samples were collected to measure VEGF-A, VEGFR1, and VEGFR2 levels using Quantikine immunoassays and other metabolic parameters such as lipid profiles and HbA1c [56]. The analysis revealed no significant differences in VEGF-A, VEGFR1, or VEGFR2 levels between the diabetic and control groups [56]. However, among T2DM patients, VEGF-A and VEGFR2 levels positively correlated with triglyceride levels, while VEGFR2 concentrations were inversely associated with HDL cholesterol [56]. These findings suggest that lipid metabolism may modulate angiogenic factors in T2DM, even without apparent vascular complications.

VEGF-B has been implicated in DKD by promoting ectopic lipid deposition in glomerular podocytes, thereby contributing to kidney damage [58]. Recent evidence underscores the critical role of VEGF-B and its receptor, VEGFR-1, in lipid metabolism dysregulation, inflammation, and the progression of DKD [59]. Transcriptomic analyses of samples from DKD patients revealed the upregulated expression of VEGF-B/VEGFR-1, accompanied by the activation of VEGF signaling pathways that facilitate ectopic lipid accumulation in renal tissues [59]. Additionally, IL-17A signaling was identified as a major contributor to inflammatory processes, further exacerbating renal injury in DKD [59]. This dual involvement of VEGF-B in lipid metabolic dysfunction and IL-17A in inflammation suggests a complex interplay between the metabolic and immune mechanisms driving DKD progression [59]. Table 2 summarizes the core findings of the discussed studies. 

### 3.4. Potential Treatment Implications 

The role of VEGF and VEGFR in DKD is multifaceted, functioning as both protective and risk factors depending on the disease context and stage. VEGF-A supports endothelial health by preserving the integrity of glomerular endothelial cells and podocytes, which is crucial for maintaining the glomerular filtration barrier and preventing dysfunction and podocyte loss [60,61]. During the early stages of DKD, VEGF-A also promotes angiogenesis, aiding tissue repair [60].

Nevertheless, excessive VEGF-A production in diabetic conditions is associated with glomerular hypertrophy, increased albuminuria, and the disruption of the filtration barrier due to podocyte dysfunction [60,61]. Anti-VEGF-A strategies, such as VEGF-A inhibitors or soluble receptors like sFlt-1, have shown promise in animal models by reducing albuminuria, glomerular hypertrophy, and mesangial expansion. For instance, in diabetic rats, suramin—a compound that influences VEGF signaling—reduced VEGFR2 mRNA expression in the renal cortex but had no significant effect on VEGFR1 levels [50]. Functionally, suramin improved endothelial relaxation in response to acetylcholine in renal arteries, partially restoring endothelial function [50]. However, suramin treatment did not significantly affect the glomerular filtration rate, serum adhesion molecules, or other nutritional parameters, indicating a limited scope of efficacy [50]. These findings suggest suramin’s potential to modulate the VEGF-A/VEGFR axis and improve endothelial function, yet its broader therapeutic impact in DKD requires further exploration [50]. The clinical translation of anti-VEGF-A therapies remains challenging. While some studies indicate that anti-VEGF-A agents, such as intravitreal bevacizumab, could exacerbate proteinuria and glomerular damage in DKD patients, others report minimal effects on renal function. Such discrepancies may reflect the dual role of VEGF-A in vascular maintenance and endothelial repair. The over-inhibition of VEGF-A has been linked to adverse effects on the glomerular structure, emphasizing the importance of developing therapies that carefully balance VEGF-A modulation to avoid unintended harm. This complexity underscores the need for nuanced therapeutic approaches targeting VEGF-A/VEGFR2 signaling in DKD management, combining efficacy with safety [62,63,64,65,66,67,68].

VEGF-B plays a critical role in renal impairment by promoting lipid accumulation in podocytes, leading to lipotoxicity and aggravating the progression of DKD. Elevated VEGF-B levels have been associated with worsening renal function markers, suggesting their potential as an independent risk factor for DKD progression [59,69]. Cao et al. investigated the therapeutic potential of neutralizing antibodies targeting VEGF-B (with anti-VEGF-B antibody) and IL-17A (with anti-IL-17A antibody) in a mouse model of DKD. The combined therapy outperformed single-agent treatments by effectively reducing the albumin-to-creatinine ratio, serum creatinine levels, and renal structural damage. Histological analyses showed a marked decrease in fibrosis markers, such as fibronectin and α-SMA, and a reduction in ectopic lipid deposition and triglyceride accumulation [59]. Additionally, this combined approach normalized proinflammatory cytokine levels, including TNF-α and IL-6, likely by suppressing NF-κB signaling. The transcriptomic profiling of treated mice further confirmed the reversal of key gene expression changes associated with DKD, particularly in lipid metabolism, inflammation, and fibrosis pathways. The study also identified the downregulation of fibroblast growth factor genes, such as FGFR2 and FGF12, mediated through PI3K-Akt signaling, indicating a novel mechanism of therapeutic efficacy [59]. These findings underscore the potential of targeting VEGF-B/VEGFR-1 as a crucial therapeutic strategy in DKD. They also highlight the synergistic benefits of concurrently inhibiting inflammatory pathways, offering a promising approach to managing DKD progression comprehensively [59]. Moreover, CSL346, a humanized monoclonal antibody targeting VEGF-B, was evaluated in a phase 2a randomized, placebo-controlled trial involving patients with T2DM, an elevated urinary albumin-to-creatinine ratio (UACR), and reduced eGFR. The trial assessed the efficacy of CSL346 at doses of 8 or 16 mg/kg over 16 weeks, with the primary endpoint being the change in UACR. Unfortunately, CSL346 did not significantly reduce UACR compared to placebo. Subgroup analyses based on the degree of kidney impairment or concurrent SGLT2 inhibitor use also failed to show any significant effect. Furthermore, biomarkers of proximal tubular injury remained unchanged with CSL346 treatment. While CSL346 was generally well tolerated, the 16 mg/kg dose was associated with a sustained increase in diastolic blood pressure, raising potential safety concerns. These findings suggest that VEGF-B inhibition via CSL346 may have limited efficacy in improving DKD markers. Further research is warranted to address these safety concerns, refine the therapeutic approach, and explore potential benefits in other patient populations or with combination therapies [58].

Yang et al. explored the protective effects of xanthine oxidase (XO) inhibition in DKD, emphasizing its role in mitigating oxidative stress and inflammation through the VEGF-NADPH oxidase (NOX) signaling pathway [70]. The study utilized a streptozotocin (STZ)-induced mouse model of DKD and high glucose (HG)-treated human glomerular endothelial cells (GECs) to evaluate febuxostat, an XO inhibitor across physical, biochemical, and molecular parameters. In the STZ-induced DKD model, mice displayed an increased kidney weight-to-body weight ratio, a urinary albumin-to-creatinine ratio, serum cystatin C levels, and oxidative stress markers. Febuxostat treatment significantly ameliorated these abnormalities without affecting blood glucose or HbA1c levels. Histological analysis showed mesangial matrix expansion and the increased expression of TGF-β1 and collagen IV in STZ kidneys, which were reduced with febuxostat administration. Additionally, febuxostat suppressed the upregulation of VEGF, VEGFR1, VEGFR3, and NOX family components in the kidneys of STZ mice. Febuxostat also restored diminished superoxide dismutase activity and reduced oxidative stress markers such as malondialdehyde and 8-hydroxy-*2′*-deoxyguanosine (8-OH-dG) staining in the kidneys. In vitro, febuxostat inhibited HG-induced increases in NOX1, NOX2, NOX4, FoxO3a phosphorylation, and intracellular reactive oxygen species (ROS) levels. These antioxidant effects depended on VEGFR1/3 signaling, as the benefits of febuxostat were reversed when VEGFR1/3 inhibitors were applied. Febuxostat also enhanced endothelial nitric oxide synthase (eNOS) phosphorylation in HG-treated GECs, countering oxidative stress; however, this effect was suppressed in the presence of VEGFR1/3 inhibitors. The study concluded that XO inhibition by febuxostat effectively ameliorates DKD by reducing oxidative stress and inflammation, primarily through a VEGFR1/3-dependent NOX-FoxO3a-eNOS signaling mechanism [70]. 

Hwang et al. investigated the therapeutic potential of SAR131675, a VEGFR-3 inhibitor, in mitigating DKD by suppressing renal lymphangiogenesis and macrophage polarization under lipotoxic conditions. In a diabetic mouse model (db/db), SAR131675 reduced systemic and renal inflammation, lipid accumulation, and albuminuria while normalizing key inflammatory markers, including MCP-1 and TNF-α levels. SAR131675 selectively inhibited VEGF-C and VEGFR-3 signaling, critical mediators of lymphangiogenesis, without affecting VEGFR-1 or VEGFR-2 expression. The treatment also attenuated glomerulosclerosis and tubulointerstitial fibrosis by reducing markers such as TGF-β1, collagen IV, and α-SMA, suggesting a protective effect on renal structure. In vitro, SAR131675 counteracted palmitate-induced lipotoxicity in human proximal tubular epithelial cells (HK2) and macrophages (RAW264.7) by suppressing VEGF-C, VEGFR-3, and LYVE-1 expression, while also inhibiting M1 macrophage polarization and oxidative stress. These findings highlight SAR131675’s ability to modulate key VEGF/VEGFR pathways in DKD, emphasizing its role in controlling lymphangiogenesis and inflammation-driven renal injury [71].

Angiogenic factors, particularly VEGF, have been explored as therapeutic targets for DKD. While VEGF inhibitors have shown benefits in diabetic ocular diseases and experimental DKD models, their clinical application remains controversial. Evidence suggests that anti-VEGF therapies may worsen proteinuria and glomerular microangiopathy in some patients, while other studies report no significant renal harm. Although VEGF/VEGFR signaling has protective roles, its dysregulation—especially regarding VEGF-B—underscores the need for careful therapeutic targeting. Targeting VEGF/VEGFR signaling presents challenges due to their systemic effects, highlighting the need for precise, tissue-specific interventions. The over- or under-inhibition of VEGF can be harmful, as evidenced by adverse effects such as proteinuria and glomerular thrombotic microangiopathy observed with anti-VEGF therapies like bevacizumab and ranibizumab. Furthermore, individual variability in disease progression and therapeutic response underscores the importance of personalized treatment approaches [43,63,64,65,66,72].

## 4. Role of the NO/NOS System in DKD

### 4.1. Overview

NO is a crucial signaling molecule with antihypertensive and renoprotective effects, primarily produced in the kidneys by three isoforms of NOS: neuronal NOS (nNOS), inducible NOS (iNOS), and endothelial NOS (eNOS). Each isoform is localized differently within the kidney and plays a distinct role in kidney physiology and pathology. Notably, nNOS is found in areas such as the Bowman’s capsule and macula densa, eNOS is concentrated in the vascular endothelium, and iNOS expression is primarily associated with pathological states [73]. These NOS isoforms facilitate essential functions, including regulating renal blood flow, renin release, renal sympathetic activity, and the tubuloglomerular feedback (TGF) system, which are fundamental to kidney health [74,75]. The TGF system modulates eGFR by inducing renin secretion, leading to vasoconstriction through the RAAS, when macula densa cells detect increased distal fluid and sodium chloride concentrations [76].

### 4.2. Animal Models

eNOS gene polymorphisms, which lead to altered eNOS transcription, have been associated with a more severe progression of DKD [77]. eNOS plays a potential role in developing DKD, as demonstrated in studies using diabetic eNOS−/− mice, which exhibit more pronounced renal morphological changes compared to diabetic eNOS+/+ mice [78]. In diabetic eNOS−/− mice, the absence of eNOS is associated with increased activity of the RAAS and elevated levels of VEGF, which contribute to changes in renal hemodynamics and morphology. Endothelial injury in these mice triggers local inflammation, platelet aggregation, mesangial expansion, and podocyte damage, all of which contribute to the progression of DKD [78].

In the early stages of diabetes, NO production is often increased, primarily mediated by the constitutively expressed nNOS and eNOS. This enhanced NO production can lead to hyperfiltration and microalbuminuria, characteristic features of early DKD [79]. As diabetes progresses, studies have shown a significant decrease in nNOS activity and kidney protein levels. For example, research involving STZ-induced diabetic rats demonstrated that nNOS expression in the kidney cortex was markedly reduced over time, which correlated with worsening renal function. This decline in nNOS activity may contribute to the transition from hyperfiltration to renal impairment [79]. The reduction in nNOS activity during the advanced stages of DKD is associated with several factors, including oxidative stress, advanced glycation end-products, and inflammatory mediators. These factors can impair the NO signaling pathway, leading to endothelial dysfunction and decreased renal blood flow [80].

In STZ-induced DKD, iNOS knockout mice exhibited lower urine NO_2_^−^ and NO_3_^−^ concentrations than controls. This suggests that iNOS may play a role in the pathogenesis of DKD, as diabetic iNOS knockout mice are more susceptible to developing severe glomerular injury and tubulointerstitial fibrosis than diabetic C57BL/6 mice. During DKD, eNOS expression is higher in iNOS knockout mice than in C57BL/6 mice [81].

### 4.3. Human Studies

In humans, increased glomerular eNOS expression has been observed in patients with type II diabetes [82]. Patients of African and Asian descent are more vulnerable to the development of end-stage kidney disease and exhibit decreased NO production in cases of DKD compared to Caucasians [83]. VEGF, IGF-1, and hyperglycemia are key factors that trigger increased eNOS expression and activity in the early stages of DKD. However, the RAAS and increased superoxide production may influence the dysfunction of eNOS and the altered bioavailability of NO as DKD progresses [84,85].

As NO is an unstable molecule, human studies on NO metabolism are typically limited to the measurement of urine and serum concentrations of its metabolites, nitrite (NO_2_^−^) and nitrate (NO_3_^−^) [86]. Reduced serum levels of NO_2_^−^ and NO_3_^−^ have been reported in patients with T2DM who developed DKD [87] and in patients with T1DM accompanied by glomerular hyperfiltration [88]. Conversely, increased serum levels of NO metabolites have been observed in patients with proteinuria and children with T1DM [89,90]. Urine levels of NO_2_^−^ and NO_3_^−^ may provide more accurate information on renal NO metabolism [88]. Sokolovska et al. were the first to measure NO concentration using electron paramagnetic resonance spectroscopy in patients with T1DM [91]. In their study, the absence of diabetes-induced kidney damage was associated with increased NO levels and decreased serum concentrations of NO metabolites. As DKD progressed, urine levels of NO_2_^−^ and NO_3_^−^ decreased, while serum levels remained comparable to those of healthy controls. Although renal morphological changes in patients with T1DM and T2DM are similar, alterations in T2DM appear to be more heterogeneous and generally do not correspond to the overall clinical presentation [92].

Genetic polymorphisms in NOS genes may influence susceptibility to DKD. Variants in the nNOS gene have been linked to altered NO production and may play a role in the development and progression of DKD [93]. Król-Kulikowska et al. explored the role of NOS isoforms and genetic polymorphisms in DKD before and after kidney transplantation. They observed lower eNOS, nNOS, and iNOS concentrations in DKD patients compared to healthy controls, with similar reductions in iNOS levels in post-transplant patients [93]. Specific genetic polymorphisms were identified as significant, such as the rs3782218 variant in nNOS, which was associated with an increased risk of developing DKD and requiring renal replacement therapy. Variations in eNOS, including rs1799983 and rs2070744, also showed differences in genotype distribution, with certain genotypes being linked to altered eNOS3 levels [93]. DKD patients also exhibited higher glucose and inflammation markers (such as CRP) and lower zinc concentrations, particularly in those with specific genetic variants. These findings highlight the interplay between NOS dysregulation, genetic predispositions, and metabolic alterations in the pathogenesis of DKD [93]. The key findings from the included human studies on the role of the NO/NOS system in DKD are summarized in Table 3.

### 4.4. Potential Therapeutic Implications

The NO/NOS pathway plays a dual role in DKD, acting as both a protective factor and a risk factor depending on the disease stage and context. In the early stages of DKD, NO produced by eNOS supports endothelial health by regulating vascular tone, inhibiting platelet aggregation, and preventing leukocyte adhesion—functions essential for maintaining kidney health [78]. NO also stabilizes renal blood flow and filtration through its role in autoregulation, counteracting the hemodynamic changes associated with diabetes [94]. However, prolonged hyperglycemia and oxidative stress reduce NO bioavailability, impairing eNOS activity and leading to endothelial dysfunction, a key driver of DKD progression [94]. In advanced stages, iNOS exacerbates chronic inflammation by producing excessive NO, further contributing to tissue damage [94]. Reduced eNOS activity and NO availability are linked to worsening kidney function, increased albuminuria, and structural renal damage, such as glomerulosclerosis [78,94].

Leonova et al. explored the effects of new 1,4-dihydropyridine (DHP) derivatives—cerebrocrast, etaftorone, and fenoftorone—on NO production and NOS expression in a diabetic rat model. Diabetes significantly increased kidney NO levels and inducible NOS iNOS expression while reducing endothelial NOS eNOS expression. Etaftorone effectively reduced NO levels, downregulated iNOS, and increased eNOS expression, helping restore balance in NO production. Additionally, etaftorone mitigated kidney damage, including glomerular edema and tubular degeneration, and decreased xanthine oxidoreductase expression, another contributor to oxidative stress. These results suggest that DHP derivatives, especially etaftorone, may help prevent kidney damage in diabetes by modulating NO and NOS pathways [95].

Given the protective role of NO, strategies to enhance nNOS activity or increase NO bioavailability could offer therapeutic benefits in DKD. For instance, treatment with L-arginine, a precursor for NO synthesis, has demonstrated promising results in mitigating glomerular hyperfiltration and reducing proteinuria in diabetic models [96]. Combining nNOS-targeting therapies with established DKD treatments, such as RAAS inhibitors, could enhance therapeutic efficacy. RAAS inhibitors already reduce glomerular pressure and inflammation, and their integration with nNOS modulators may yield synergistic renal protective effects [79,80]. Furthermore, emerging pharmacological agents designed to enhance nNOS activity or mimic its effects selectively may represent a new avenue for DKD therapy, potentially restoring renal hemodynamics and counteracting diabetes-induced renal damage [79,80]. Thus, maintaining NO bioavailability while addressing its dysfunction is crucial for effectively managing DKD.

## 5. Limitations

The current review highlights significant gaps in our understanding of the apelinergic system, VEGF/VEGFR signaling, and NO/NOS pathways in DKD. Human studies on these molecular pathways are limited and often produce inconsistent results compared to preclinical findings. The dual roles of these pathways, which can both protect and damage renal function depending on the context, pose challenges for therapeutic development. For example, while apelin and VEGF exhibit renoprotective effects in some cases, their dysregulation can worsen kidney damage, complicating efforts to design targeted interventions. Safety concerns, such as the potential for anti-VEGF therapies to exacerbate proteinuria or glomerular damage, further hinder clinical applications. Additionally, DKD’s heterogeneity, influenced by genetic, metabolic, and disease-specific factors, underscores the need for personalized treatment strategies. Addressing these limitations requires expanded clinical studies, detailed mechanistic research, and the integration of new molecular therapies with established treatments like RAAS and SGLT2 inhibitors to improve outcomes while minimizing risks.

## 6. Conclusions

The intricate interplay between the apelinergic system, VEGF/VEGFR pathways, and NO/NOS signaling highlight their critical roles in the pathogenesis and potential treatment of DKD. The apelinergic system demonstrates renoprotective effects through anti-inflammatory and antioxidant mechanisms, though conflicting evidence suggests it may exacerbate DKD in specific contexts. VEGF/VEGFR signaling, particularly involving VEGF-A and VEGFR-2, is pivotal in regulating angiogenesis and vascular integrity, yet its dysregulation contributes to pathological angiogenesis, fibrosis, and renal damage. NO/NOS signaling, crucial for renal vascular health, is often disrupted in DKD, with alterations in NO bioavailability and endothelial dysfunction driving disease progression.

Despite promising preclinical findings, human studies reveal variable outcomes, underscoring the complexity of these pathways and the challenges in translating findings into effective therapies. Anti-VEGF strategies, apelin receptor modulation, and NO-based interventions hold potential but require careful balancing to minimize adverse effects, such as exacerbated proteinuria or impaired podocyte function. Furthermore, individual variability in disease progression and response to treatment necessitates a personalized approach to DKD management.

Ongoing research should aim to refine therapeutic strategies targeting these pathways, focusing on their integration with existing treatments like RAAS inhibitors. Addressing the gaps in understanding their roles across different stages of DKD and patient populations will be crucial in advancing treatment outcomes and reducing the burden of this debilitating condition.

## Figures and Tables

**Table 1:** Summary of human studies on the apelinergic system in diabetic kidney disease (DKD). T1DM: type 1 diabetes mellitus; T2DM: type 2 diabetes mellitus.

Study [Reference]	Patients (n)		Results
	Diabetic Patients	Controls	
Habchi et al. [21]	130 T1DM 98 T2DM	162	Serum apelin levels are higher in diabetic patients compared to non-diabetic controls. Additionally, serum apelin levels are elevated in patients with T1DM compared to those with T2DM.
Zheng et al. [22]	498	1468	A significant correlation was observed between the expression of the apelin rs2281068 gene and diabetes in patients from the Chinese population when compared to non-diabetic controls.
Kotanidou et al. [23]	90	90	Obese patients with impaired glucose metabolism (diabese) exhibited lower apelin levels compared to controls.

**Table 2:** Summary of human studies on the role of vascular endothelial growth factor (VEGF)/ vascular endothelial growth factor receptor (VEGFR) system in diabetic kidney disease (DKD). mRNA—Messenger Ribonucleic Acid.

Study [Reference]	Patients (n)		Results
	Diabetic Patients	Controls	
Lindenmeyer et al. [52]	10	3	VEGF-A levels are decreased in patients with DKD compared to healthy controls.
Mahdy et al. [53]	55	15	Serum VEGF levels are significantly higher in diabetic patients compared to healthy controls.
Kubisz et al. [54]	84	42	A significant increase in serum VEGF levels has been observed in diabetic patients compared to healthy controls.
Ruszkowska-Ciastek et al. [56]	31	30	No significant differences were found in the concentrations of VEGF-A, VEGFR1, or VEGFR2 between the diabetic group and the control group.
Loewen et al. [57]	918 DKD biopsies	Kidney biopsies without histological alterations in light and immunohistochemistry microscopy were taken as controls.	Elevated levels of VEGF-A mRNA are associated with the formation of aberrant arterioles in the glomeruli of patients with DKD. Additionally, VEGFR-2 expression is increased in diabetic patients compared to healthy controls.

**Table 3:** Summary of human studies on the role of nitric oxide (NO)/ nitric oxide synthase (NOS) system in diabetic kidney disease (DKD). NOx—NO_2_^−^/NO_3_^−^ (NO products) excretion; T1DM—type 1 diabetes mellitus.

Study [Reference]	Patients (n)		Results
	Diabetic Patients	Controls	
Hiragushi et al. [76]	82	84	Urinary NOx excretion is increased in diabetic patients compared to controls.
Tessari et al. [82]	8	10	Intravascular NOx synthesis is reduced in diabetic patients compared to healthy controls.
Cherney et al. [83]	37	21	In diabetic patients, NO bioactivity is increased in the kidneys but decreased in the systemic circulation.
Sokolovska et al. [86]	268	69	Patients with T1DM without DKD have higher blood NO levels and lower serum NO₂⁻ and NO₃⁻ levels compared to controls. However, there is no significant difference in the serum levels of NO₂⁻ and NO₃⁻ between patients with T1DM and DKD compared to controls.
Król-Kulikowska et al. [88]	182	50	eNOS concentrations are lower in patients with DKD compared to healthy controls.

## Data Availability

Not applicable.
